# Association between antipsychotic drug dose and length of clinical notes: a proxy of disease severity?

**DOI:** 10.1186/s12874-020-00993-1

**Published:** 2020-05-07

**Authors:** Freja Karuna Hemmingsen Sørup, Søren Brunak, Robert Eriksson

**Affiliations:** Disease Systems Biology program, Novo Nordisk Foundation Center for Protein Research, University of Copenhagen, Blegdamsvej 3B, DK-2200 Copenhagen N, Denmark

**Keywords:** Adverse event, Text mining, Natural language processing, Antipsychotic drugs

## Abstract

**Background:**

Most structured clinical data, such as diagnosis codes, are not sufficient to obtain precise phenotypes and assess disease burden. Text mining of clinical notes could provide a basis for detailed profiles of phenotypic traits. The objective of the current study was to determine whether drug dose, regardless of polypharmacy, is associated with the length of clinical notes, and to determine the frequency of adverse events per word in clinical notes.

**Methods:**

In this observational study, we utilized restricted-access data from an electronic patient record system. Using three methods (defined daily dose, olanzapine equivalents, and chlorpromazine equivalents) we calculated antipsychotic dose equivalents and compared these with the number of words recorded per treatment day. For each normalization method, the frequencies of adverse events per word in manually curated samples were compared to dose intervals.

**Results:**

The length of clinical notes per treatment day was positively associated with the prescribed dose for all normalization methods. The number of adverse events per word was stable over the analyzed dose spectrum.

**Conclusions:**

Assuming that drug dose increases with the severity of disease, the length of clinical notes can serve as a proxy for disease severity. Due to the near-linear relationship, correction of daily word count is unnecessary when text mining for potential adverse drug reactions.

## Background

Currently, drug safety surveillance efforts rely heavily on spontaneous reporting systems for post-approval monitoring [1]. However, such spontaneous reports suffer from a variety of issues, including massive under-reporting [2], and therefore alternative real-world data approaches are being developed. One of these approaches is to monitor adverse events extracted from clinical narratives by text mining [3] and we have previously created a text-mining pipeline for this specific purpose [4, 5]. In order to develop efficient text mining approaches to investigate adverse events, a range of obstacles needs to be addressed and causes of systemic biases identified.

Safety monitoring is further complicated by polypharmacy and the fact that drugs may be used in higher doses than recommended in guidelines [6], both of which are associated with adverse drug reactions as well as disease severity [7, 8]. Antipsychotics are a drug class associated with frequent polypharmacy in the treatment of seriously ill psychiatric patients [9, 10]. However, uncovering any association between a specific characteristic and antipsychotic dose load is complicated by the difficulty of comparing drugs within the drug class. To facilitate comparisons between different antipsychotics, several methods for calculating antipsychotic equivalents have been suggested [11–13] and it has been argued that none of the methods is superior or should be considered the gold standard [14]. By converting all antipsychotic drugs to equivalents, polypharmacy can be converted to one single equivalent dose and enable comparisons.

Electronic patient records have emerged as a powerful documentation and communication resource in healthcare systems. These records have been shown to reflect processes and structures within healthcare systems, and this might be important to consider when using clinical data for research purposes [15]. Such processes could potentially introduce study biases, or it could be that structural components of the record could be used as proxies for specific clinical variables, for instance disease severity or mortality.

The current study sought to explore whether the drug dose load is associated with the length of the clinical notes. The analysis was performed on three subsets: All notes recorded on the patient, notes recorded by physicians, and notes recorded by nursing staff. Further, we aimed to investigate whether the frequency of potential adverse events per word was influenced by drug dose load. Such associations might influence text-mining efforts through systemic biases, and might therefore require some form of normalization based on the dose each patient receives, or alternatively the number of words in the record.

## Methods

### Study population

This study is based on clinical narratives and structured prescription data from patients admitted to a Danish tertiary mental health center in the period January 2000 to June 2010. All patients treated with a minimum of one antipsychotic drug fulfilled the inclusion criteria. We required the antipsychotic dosing data to be comprehensive. This meant that we excluded all patients where the prescription data could not be unambiguously ascertained. Furthermore, we excluded patients from each subanalysis if we could not calculate an equivalent for one or more treatment days.

### Patient characteristics

We determined the distribution of sex, mean age and the number of diagnoses in each of the groups created based on the three normalization methods. All diagnoses had been assigned the appropriate International Classification of Diseases version 10 codes [16] (ICD-10) by the hospital.

### Antipsychotic equivalents

The patients received a wide range of antipsychotic drugs, both as monotherapy and as polypharmacy. To enable comparison of daily drug exposures we used three methods: defined daily dose (DDD), [11] chlorpromazine equivalents [12], and olanzapine equivalents [13]. The total daily antipsychotic equivalent for each patient and day were summed.

### Clinical narratives and dose

In the study we used the daily word count to represent the length of the clinical notes. The notes were extracted from the medical narratives section of the electronic patient records. We used the Unix command wc to count words. The total word count for each treatment day was summed to form these daily word counts. We created three groups of notes to compare whether the recording authors’ profession had an influence: Firstly, one category containing all clinical notes regardless of the authors’ profession. Secondly, notes recorded by physicians. Thirdly, notes recorded by nursing staff.

**Fig. 1:**
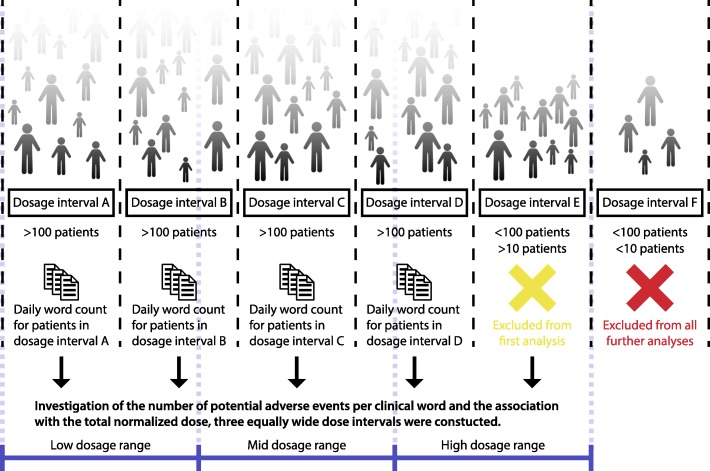
Initially dose interval groups were formed by binning equivalent doses. The three equivalents consisted of separate dosage intervals, all starting from 0. DDDs were binned in intervals of 0.5 DDD, chlorpromazine equivalents were binned in intervals of 100 mg, and olanzapine equivalents were binned in intervals of 5 mg. The length of the clinical notes was analyzed in each binned dosage interval. Three equally wide dose intervals (low, mid, high) were defined to investigate whether the number of potential adverse events per clinical word was associated with the total normalized dose. Intervals containing less than 10 patients were excluded from all analyses, intervals containing less than 100 patients were only excluded from the analysis comparing the drug dose with the length of clinical notes

All daily equivalent doses were binned into dose interval groups. The intervals were defined as starting from 0 and binning DDDs in intervals of 0.5 DDD, chlorpromazine equivalents in intervals of 100 mg, and olanzapine equivalents in intervals of 5 mg. The lower boundary of each interval was greater than the cut-off value and the upper boundary was equal to the cut-off value (Fig. 1).

For each treatment day considered, a patient contributed with a daily equivalent dose and a medical record word count based on all notes recorded on that day. We calculated the average word count per day for each patient by averaging the word count per treatment day, for all days on which the patient’s daily equivalent dose was within the interval of each bin. To explore the association between antipsychotic dose load and number of words per day, the median for each bin was compared, and the distribution of each interval for all three methods of dose normalization was plotted. Intervals containing less than 100 patients were excluded from the analysis.

### Influence of drug dose on the potential adverse events per word

To investigate whether the number of potential adverse events per clinical word was associated with the total normalized dose, three equally wide dose intervals for each normalization method were defined. The three intervals for each normalization method were chosen to include the broadest spectrum of doses, based on the previously described binned dose intervals containing ten or more patients, and the groups therefore spanned a range of bins (Fig. 1). We manually curated all records from 125 randomly selected treatment days in each of the dose intervals, multiple records were allowed to originate from the same patient. All potential adverse events were compared to the total amount of words recorded in the clinical narratives.

## Results

In total 2838 patients fulfilled the inclusion criteria. Of these 1249 patients were excluded, meaning 1589 patients were included in the analyses. Only the DDD normalization method [11] held conversions for all antipsychotic drugs in all the formulations received by our study population. The olanzapine equivalent method [13] includes 19 out of 21 drugs and the chlorpromazine equivalent method [12] includes 9 out of 21 drugs (Table 1). Since we required certainty in dose calculations, there are fewer patients in the analyses using the olanzapine and chlorpromazine normalization methods; patient characteristics also differ (Table 2). The most common diagnosis across normalization methods was schizophrenia.

**Table 1:** Drugs covered by the conversion methods

Drug	DDD	Olanzapine equivalents	Chlorpromazine equivalents
**amisulpride**	YES	YES	NO
**aripiprazole**	YES	YES	YES
**chlorprothixene**	YES	YES	NO
**clozapine**	YES	YES	YES
**flupentixol**	YES	YES	NO
**haloperidol**	YES	YES	YES
**levomepromazine**	YES	YES	NO
**melperone**	YES	NO	NO
**olanzapine**	YES	YES	YES
**paliperidone**	YES	YES	NO
**penfluridol**	YES	NO	NO
**perphenazine**	YES	YES	YES
**pimozide**	YES	YES	NO
**prochlorperazine**	YES	YES	NO
**quetiapine**	YES	YES	YES
**risperidone**	YES	YES	YES
**sertindole**	YES	YES	NO
**sulpiride**	YES	YES	NO
**thioridazine**	YES	YES	YES
**ziprasidone**	YES	YES	YES
**zuclopenthixol**	YES	YES	NO

**Table 2:** Patient characteristics for the cohorts covered by the three normalization methods. Diagnoses are coded in International Classification of Diseases version 10

Normalization method	DDD n (%)	Olanzapine equivalents n (%)	Chlorpromazine equivalents n (%)
Number of patients	1589	1539	438
Male sex	1043 (65.6)	1010 (65.6)	287 (65.5)
Mean age in years (SD)	40.3 (6.3)	40.1 (6.4)	40.1 (6.3)
**Diagnoses**			
F10 Alcohol related disorders	666 (41.9)	645 (41.9)	149 (34.0)
F11 Opioid related disorders	266 (16.7)	256 (16.6)	66 (15.1)
F12 Cannabis related disorders	476 (30.0)	458 (29.8)	130 (29.7)
F13 Sedative, hypnotic, or anxiolytic related disorders	255 (16.0)	247 (16.0)	57 (13.0)
F14 Cocaine related disorders	189 (11.9)	182 (11.8)	41 (9.4)
F15 Other stimulant related disorders	126 (7.9)	122 (7.9)	28 (6.4)
F19 Other psychoactive substance related disorders	225 (14.2)	218 (14.2)	60 (13.7)
F20 Schizophrenia	740 (46.6)	707 (45.9)	197 (45.0)
F21 Schizotypal disorder	105 (6.6)	103 (6.7)	28 (6.4)
F22 Delusional disorder	56 (3.5)	54 (4.9)	20 (4.6)
F23 Brief psychotic disorder	25 (1.6)	25 (1.6)	10 (2.3)
F25 Schizoaffective disorder	50 (3.1)	49 (4.9)	13 (3.0)
F31 Bipolar disorder	98 (6.2)	97 (6.3)	26 (5.9)
F32 Mayor depressive disorder, single episode	120 (7.6)	119 (7.7)	18 (4.1)
F33 Mayor depressive disorder, recurrent	197 (12.4)	193 (12.5)	40 (9.1)
F41 Other anxiety disorders	162 (10.2)	159 (10.3)	33 (7.5)
F60 Specific personality disorders	329 (20.7)	325 (21.1)	80 (18.3)
Z046 Encounter for general psychiatric examination, requested by authority	266 (16.7)	250 (16.2)	66 (15.1)

**Fig. 2:**
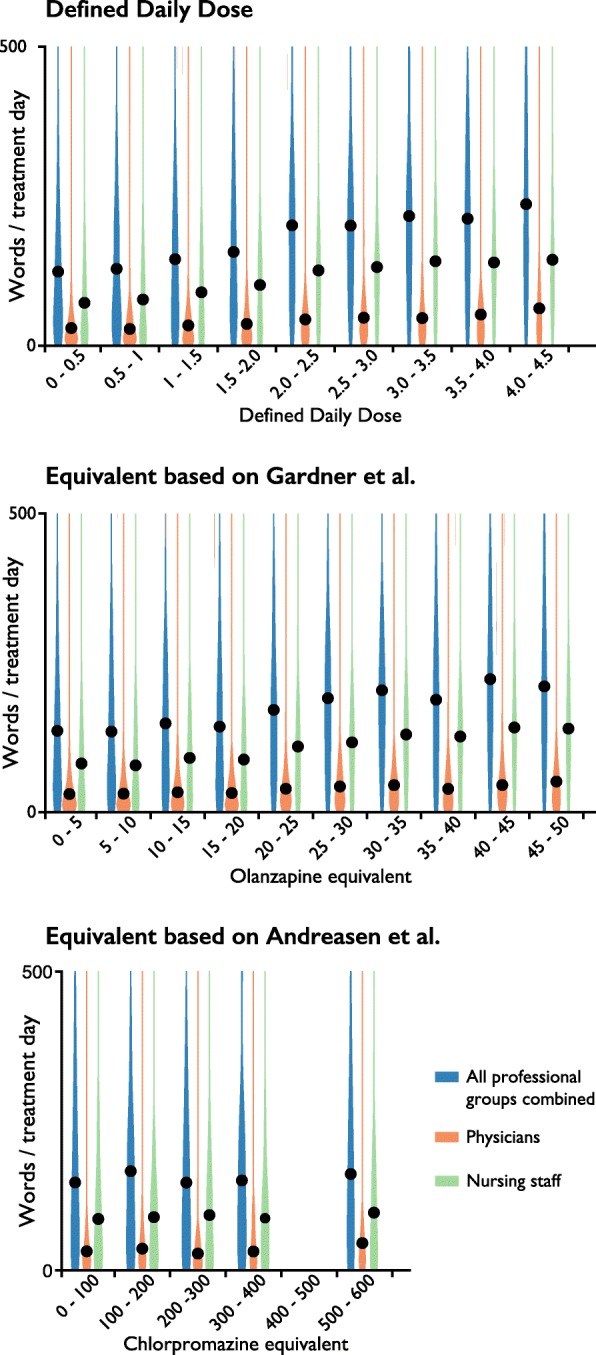
Violin plots of antipsychotic dose load and number of words in the clinical notes per day using the three equalization methods. The medians of the distributions are represented by black dots. The width of each area represents a dose interval of, respectively, 0.5 DDD, 5 mg olanzapine equivalents, or 100 mg chlorpromazine equivalents. The same intervals were used to bin data from notes recorded by all staff categories (physicians, nursing staff, physical therapists, occupational therapists, psychologists, social workers, and secretaries), notes by physicians, and notes by nursing staff. The daily note length by all staff, physicians and nursing staff are plotted individually. Each value of the note length originates from zero and the values are not additive. Intervals containing less than 100 patients are not plotted

In total 4,903,669 notes were stored in the patient records; of these, physicians had recorded 885,964 (18%) notes and nursing staff had recorded 3,726,529 (76%) notes. We found a positive association between the number of clinical note words per day and prescribed dose for all normalization methods, irrespective of the staff category recording the note (Fig. 2).

**Fig. 3:**
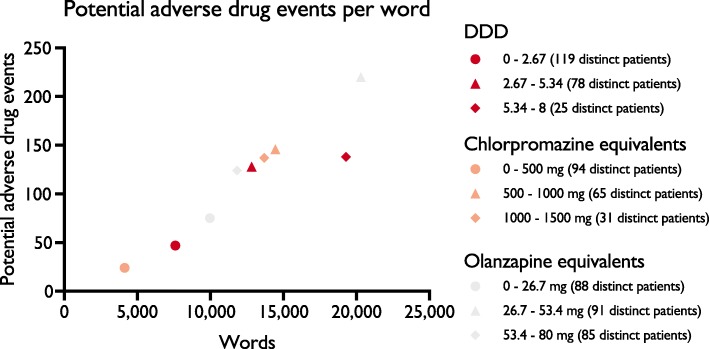
Potential adverse drug events per word recorded in the clinical narratives. Three dose intervals were selected for each of the three normalization methods

Three intervals were chosen to determine potential adverse events per treatment day and the numbers of potential adverse events per word were plotted for the three normalization methods. The number of patients included in the intervals spanned between 25 and 119. (Fig. 3). The average potential adverse events per word were determined to 0.0078 (DDD), 0.0086 (chlorpromazine equivalents), and 0.0096 (olanzapine equivalents).

## Discussion

Adverse drug reactions are highly underreported and searching for adverse events mentioned in patient records might increase our chance of discovering adverse drug reactions experienced by patients. When extracting adverse events, it is important to limit systemic biases. In the current study we were able to identify a positive association between the length of clinical notes and drug load. These findings were consistent in two of the normalization methods used, as well as across professions examined in this study. Likewise, consistently across normalization methods, we found a near-linear relationship between number of words in clinical notes and potential adverse events.

We performed one analysis of dose and words with all staff categories included. In addition, we analyzed two subgroups (physicians and nursing staff). The remaining staff (physical therapists, occupational therapists, psychologists, social workers, secretaries) together contributed 6% of the notes. Subgroup analyses of the remaining staff categories were not preformed due to the small number of notes within each category. Physicians and nursing staff are also the primary groups involved in pharmacological treatment.

We used three different antipsychotic drug dose normalization methods, where two methods included only some of the antipsychotic drugs taken by our patient group, resulting in three patient cohorts. One of these methods, normalizing by chlorpromazine equivalents, had so few conversions that almost three quarters of the original patients were excluded. This resulted in very few patients in the designated bins, representing mainly the very low end of daily doses expected in a clinical setting. The results for the two other normalization methods are consistent and span broader daily dose ranges.

Assuming that the patients in the data set who are most severely ill also receive higher drug doses, our results suggest that length of the daily narratives could be used as a proxy for disease severity. The number of words per day could be used for stratifying patients, as the number of words would serve as a predictor of disease severity. However, in the current study we have not compared disease severity with dose and a disease severity classification would be out of scope of the current study. We consider alternatives such as analyses of disease severity through diagnosis codes or number of diagnoses to be insufficient. We deem it impossible to completely establish disease severity from all ICD-10 diagnosis codes and a higher number of diagnoses does not necessarily mean a patient is more ill. The former, is exemplified by several diseases only having one severity level, such as “paranoid schizophrenia” (ICD-10 code F20.0). The latter, could be exemplified by most clinicians would consider a single schizophrenia diagnosis code to be worse than “acute nasopharyngitis” (ICD-10 code J00.0) diagnosis code in combination with “problems in relationship with parents and in-laws” (ICD-10 code Z63.1).

Previous research has focused on duplication [17] or redundancy [18] in patient records, but to our knowledge, this is the first time someone has reported a possible association between number of words per day and a drug treatment. The higher number of words per treatment day could depend on various factors. We hypothesize that patients prescribed higher doses have more severe disease forms, receive more involuntary treatment, are prescribed antipsychotic polypharmacy and experience more adverse drug reactions. Any of these would explain the need for more documentation and thus more words in the clinical record, which also serves as a legal document, and in some countries, for reimbursement purposes. However, when examining the possible association between number of words and possible adverse events in the narratives we find a linear relation with a constant number of events per word. It therefore seems like there is no need for adjustment for the number of words in the clinical narratives when text mining for possible adverse drug events since the results suggest that the proportion between these two variables is constant for all doses. Since the relationship is constant we suggest that no correction factor is needed to counteract effects from differences in note length. More adverse events are likely experienced at higher dose levels, as the notes recorded about patients receiving higher doses are longer and therefore contain more potential adverse events.

Since the dose analyses are performed by an algorithm there is a risk of misclassification that would have been identified with manual review. This risk exists in both the dose identification as well as the adverse event identification. In addition to these limitations, it is also a possibility that the daily dose load is not being calculated correctly. We present findings that are consistent in the normalization methods but still there is a risk of these methods not producing an accurate estimate of total daily dose. Finally, the use of data from a single center is a limitation and the discovered potential bias might be associated with care delivery at this specific unit.

## Conclusions

The prescribed drug dose is positively associated with the number of words recorded per day in the clinical notes, regardless of the staff category recording the notes. This means that the length of clinical notes in terms of word count might serve as a proxy for disease severity, assuming that drug dose increases along with disease severity. The number of potential adverse events per word in the clinical notes is close to linear and in text mining efforts of potential adverse events per day no correction of note length seems necessary.

## Data Availability

No part of the restricted-access patient records will be made public due to their sensitive nature, as the identity of the patients may be compromised if the narrative data is shared.

## Abbreviations

DDD: Defined Daily Dose
ICD-10: International Classification of Diseases version 10
